# Reply to White and Siegrist, “Multi-drug-resistant enterococcal infections: duration of combination therapy and OPAT challenges”

**DOI:** 10.1128/aac.01785-25

**Published:** 2026-03-16

**Authors:** Cesar A. Arias, Truc T. Tran, Paul Kinsella, Benjamin P. Howden, William R. Miller

**Affiliations:** 1 Division of Infectious Diseases, Department of Medicine, Houston Methodist Hospital 23534 , Houston, Texas, USA; 2 Center for Infectious Diseases, Houston Methodist Research Institute 167626 , Houston, Texas, USA; 3 Department of Medicine, Weill Cornell Medical College 12295 , New York, New York, USA; 4 Department of Microbiology and Immunology, The University of Melbourne at the Peter Doherty Institute for Infection and Immunity 534133 , Melbourne, Australia; 5 Microbiology Department, Royal Melbourne Hospital 90134, Melbourne, Australia; 6 Department of Infectious Diseases and Immunology, Austin Health 3805, Heidelberg, Australia; University of California San Francisco, San Francisco, California, USA

## REPLY

We thank White and Siegrist for their interest in our manuscript that reviews the challenges of difficult-to-treat enterococcal infections (1). The authors wanted to highlight important issues on the duration of combination therapy and challenges with outpatient parenteral therapy (OPAT).

The first point that White & Siegrist raised relates to the duration of combination therapy. We essentially agree with the authors that data are even more limited to make recommendations on the duration of combination therapy in bloodstream infections caused by multidrug-resistant *Enterococcus faecium*, particularly in the type of patient population often afflicted by vancomycin-resistant enterococcal (VRE) infections (immunocompromised individuals who often have undergone cancer chemotherapy for hematological malignancies, bone marrow transplant, or recipients of liver transplant). Thus, we focused on providing some guidance on high-risk patients (Figure 1 in Turner et al.) (2) for initial therapy, and we purposely avoided making strong statements on the duration of combination therapy. An individualized approach should be taken for each clinical scenario to determine if source control is achieved (a major issue in intra-abdominal infections after liver transplant), the presence of infective endocarditis or suspected endovascular infection (more frequently identified with *E. faecalis* than *E. faecium*), among others.

With the above issues in mind, we feel it may be reasonable to de-escalate to monotherapy once the bacteremia is cleared, source control is achieved, and endovascular foci have been ruled out. Interestingly, previous pharmacokinetic/pharmacodynamic models suggest that the activity of daptomycin (DAP) against *E. faecium* strains with MICs below the susceptible dose-dependent range is not only concentration-dependent but also appears to be inoculum-dependent (3). Indeed, in simulated endocardial vegetation models, DAP monotherapy appeared to be effective at high doses at lower inoculum (ca. 10^7^ CFU/g), supporting the de-escalation strategy once the bacterial load falls below a critical threshold. These data are consistent with the notion that an individualized approach is warranted, and the duration of therapy may vary according to the clinical scenario. Of note, we avoided using the term “complicated” bacteremia since definitions for this scenario are heterogeneous and difficult to standardize. Instead, we opted for a more patient-centric approach where the risk of poor outcomes is determined by individual patient characteristics.

We also want to point out that the combination of DAP plus ampicillin might be useful for strains that have MICs above the current CLSI proposed breakpoints (>4 µg/mL) due to the robust see-saw effect that has been documented *in vitro* and *in vivo* (4). In this scenario, a more cautious approach should be taken since it is likely that DAP exposures would be grossly suboptimal even at higher doses (10–12 mg/kg), and combination therapy may be required for the entire duration of the treatment course. Again, robust clinical data to support these assumptions are lacking.

The second issue raised by White & Siegrist is the practical approach for OPAT when ampicillin is used as part of a combination regimen. As they mention in their letter, combination therapy in which one of the drugs is ampicillin is currently the standard of care for *E. faecalis* endocarditis, and such therapy is provided using OPAT strategies despite the shortcomings of ampicillin related to stability and frequent dosing. As we discussed above, the duration of therapy should be tailored to each clinical scenario. Moreover, patients with *E. faecium* infections are often hospitalized with multiple co-morbidities, often require prolonged hospitalizations, and often complete the course of treatment. Notably, *in vitro* and *ex vivo* studies have shown that combining DAP with ceftaroline or ertapenem can be effective against *E. faecium* (3, 5, 6). These β-lactams may help circumvent the stability and administration challenges associated with ampicillin. However, ceftaroline and ertapenem have not consistently demonstrated synergistic activity across all *E. faecium* strains. Therefore, we did not prioritize them as first-line β-lactam options (Figure 1 in Turner et al.) (4–6). The role of oral agents with *in vitro* activity (e.g., linezolid, tedizolid, and omadacycline) or long-acting lipoglycopeptides such as oritavancin to complete the course of enterococcal bacteremia after an initial course of intravenous antibiotics is another area that requires further investigation and likely would vary according to the patient’s condition. For *E. faecalis* endocarditis, in areas where the administration of ampicillin is not logistically possible, the use of penicillin infusion, which is also expected to be synergistic with ceftriaxone, may represent an alternative strategy. However, no data are currently available for this approach, and the recent emergence of the penicillin-resistant ampicillin-susceptible phenotype in *E. faecalis* (7) suggests that this potential approach should be evaluated carefully.

In summary, we appreciate the thoughtful points raised by White and Siegrist. Their questions highlight the challenges posed by multidrug-resistant enterococci and the paucity of clinical data available to support specific approaches.
